# Website Quality, Expectation, Confirmation, and End User Satisfaction: The Knowledge-Intensive Website of the Korean National Cancer Information Center

**DOI:** 10.2196/jmir.1574

**Published:** 2011-11-02

**Authors:** Chulmo Koo, Yulia Wati, Keeho Park, Min Kyung Lim

**Affiliations:** ^1^College of BusinessChosun UniversityGwangju-siRepublic of Korea; ^2^Sam M. Walton College of BusinessUniversity of ArkansasFayetteville, ARUnited States; ^3^National Cancer Information CenterNational Cancer Control InstituteNational Cancer CenterGoyang-siRepublic of Korea

**Keywords:** eHealth, website quality, end user satisfaction, expectation-confirmation theory, health informatics

## Abstract

**Background:**

The fact that patient satisfaction with primary care clinical practices and physician-patient communications has decreased gradually has brought a new opportunity to the online channel as a supplementary service to provide additional information.

**Objective:**

In this study, our objectives were to examine the process of cognitive knowledge expectation-confirmation from eHealth users and to recommend the attributes of a “knowledge-intensive website.”. Knowledge expectation can be defined as users’ existing attitudes or beliefs regarding expected levels of knowledge they may gain by accessing the website. Knowledge confirmation is the extent to which user’s knowledge expectation of information systems use is realized during actual use. In our hypothesized research model, perceived information quality, presentation and attractiveness as well as knowledge expectation influence knowledge confirmation, which in turn influences perceived usefulness and end user satisfaction, which feeds back to knowledge expectation.

**Methods:**

An empirical study was conducted at the National Cancer Center (NCC), Republic of Korea (South Korea), by evaluating its official website. A user survey was administered containing items to measure subjectively perceived website quality and expectation-confirmation attributes. A study sample of 198 usable responses was used for further analysis. We used the structural equation model to test the proposed research model.

**Results:**

Knowledge expectation exhibited a positive effect on knowledge confirmation (beta = .27, P < .001). The paths from information quality, information presentation, and website attractiveness to knowledge confirmation were also positive and significant (beta = .24, P < .001; beta = .29, P < .001; beta = .18, P < .001, respectively). Moreover, the effect of knowledge confirmation on perceived usefulness was also positively significant (beta = .64, P < .001). Knowledge expectation together with knowledge confirmation and perceived usefulness also significantly affected end user satisfaction (beta = .22 P < .001; beta = .39, P < .001; beta = .25, P < .001, respectively).

**Conclusions:**

Theoretically, this study has (1) identified knowledge-intensive website attributes, (2) enhanced the theoretical foundation of eHealth from the information systems (IS) perspective by adopting the expectation-confirmation theory (ECT), and (3) examined the importance of information and knowledge attributes and explained their impact on user satisfaction. Practically, our empirical results suggest that perceived website quality (ie, information quality, information presentation, and website attractiveness) is a core requirement for knowledge building. In addition, our study has also shown that knowledge confirmation has a greater effect on satisfaction than both knowledge expectation and perceived usefulness.

## Introduction

### Background

The Internet is rapidly growing and is increasingly used as an open, anonymous, and democratic source of health information and knowledge [1]. Several studies (eg, [2]) have reported that a large percentage of the population now refers to the Internet to find health-related information as their self-reference [3]. The fact that patient satisfaction with primary care clinical practices and physician-patient communications has decreased gradually has brought a new opportunity to the online channel as a supplementary service to provide additional information [4]. This trend, known as electronic health care (eHealth), has changed the way people search for health-related information. Here, eHealth is defined as the use of the Internet to deliver access to health care information, commerce, clinical care, and other health services [5]. According to Eysenbach [6],

eHealth is an emerging field in the intersection of medical informatics, public health,and business, referring to health services and information delivered or enhanced through the Internet and related technologies. In a broader sense, the term characterizes not only a technical development, but also a stateofmind, a way of thinking, an attitude, and a commitment for networked, global thinkingto improve health care locally, regionally, and worldwide by using information and communication technology.

According to the above definition, the knowledge of what consumers find as satisfactory information in the health context has great implications, as customers may act seriously upon this information [3]. On the other hand, observational studies find that Internet users often pay little attention to source credibility when seeking out health information on the Web [1], and a lot of available information is of poor quality [3,7]. Therefore, to ensure that the best and the most accurate, timely, and relevant information is used by consumers, health care organizations have an obligation to attract users to this information [1]. Even though the importance of interactivity in website design is well recognized, studies to understand the benefits of interactive websites to attract customers are rarely developed [8]. In this regard, the information system discipline has been called to develop theories and methods that should prove the usefulness of information in eHealth [9]. Among the limitations of eHealth literature from the information systems (IS) perspective, is that there is no in-depth participatory design research on hospital or health care websites [10], and research is still lacking on the design features and development practices of consumer health information websites [11].

The main purpose of this study was to theorize the attributes of ”knowledge-intensive websites” based on the expectation-confirmation theory (ECT) and integrate these with eHealth from prior IS research. In order to maximize the function of websites as knowledge and information sources, we empirically measure website effectiveness by emphasizing the information as knowledge elements of eHealth services in that the benefits of health care are highly associated with the intrinsic value of information [12]. Secondly, we study patients’ and end users’ online behavior and investigate factors affecting their satisfaction with information provided by health care websites from the information systems perspective. This study adopted ECT to examine the process of cognitive knowledge expectation-confirmation from eHealth users. Even though ECT is widely used in marketing literature to study customer satisfaction (eg, [13]), service marketing (eg, [14]), and information systems (eg, [15-17]), few studies have employed this theory for eHealth services.

To achieve our purposes, we carried out an empirical study at the National Cancer Center (NCC), Republic of Korea (South Korea), by evaluating its official website. By considering the fact that among people with cancer, the Internet has become a major source of health information (eg, [18]), the chosen website is appropriate to our proposed model of a knowledge-intensive website. In addition, as a government funded institution, NCC has a website that may be used to evaluate acceptable criteria of a knowledge-intensive website.

The research reported here also makes several contributions to both research and practice. From a theoretical perspective, we presented the concept of a knowledge-intensive website for eHealth. We proposed and validated a range of criteria needed to establish the knowledge-based website as a main information source for patients and/or Internet users. Second, it extends the ECT in the eHealth context to explain how initial knowledge expectation together with website quality influence knowledge confirmation as an actual knowledge outcome gained by users after assessing the information and how these factors influenced postconsumption expectations that may lead to improved consumer satisfaction, which has not been examined in previous literature. Third, this paper focused on the importance of information and knowledge of an eHealth website, which is a new paradigm in the eHealth research area.

This paper is organized as follows: We begin by presenting the basic concept of expectation confirmation theory. In the third section, we describe our research model and hypotheses development. In the fourth section, we provide a description of the methodology that we relied upon to select and analyze the data, and in the fifth section, we present the results of data analysis. The sixth section presents the discussion of the study’s key findings and its limitations. And in the last section, implications and future research are discussed.

### Expectation Confirmation Theory (ECT)

The expectancy confirmation paradigm is primarily cognitive in nature because the comparison process in confirmation judgments requires the deliberate processing of information [14]. Like the original process of expectation-confirmation in explaining behavior intention, we show that the framework of actual knowledge confirmation begins with individuals’ initial expectations of a specific knowledge they may gain prior to the searching process. Thus, individuals accept and use the new knowledge. Following a period of initial consumption, individuals form perceptions about the performance or the website, that is, whether it can improve their knowledge or not. Furthermore, they assess the perceived performance of a website compared with their original expectations and determine the extent to which their expectations are confirmed. Because customers’ expectations and perceptions of performance can vary from one to another, confirmation can be positive when actual performance is higher than expectations. In this case the consumer is satisfied. But confirmation can be negative when perceived performance falls short of expectations, and, in this case, the consumer will be dissatisfied [19]. In turn, this level of satisfaction or dissatisfaction will influence intended behavior [15].

Rust et al [20] posit that customer expectations are viewed as distributions, that is, each customer has a probability density function that describes the relative likelihood that a particular quality outcome will be experienced. The satisfaction literature suggests that customers may use different types of expectations when forming opinions about a product’s anticipated performance. However, the concept of expectations has raised debate among the scholars. First, the definitions of expectations vary, ranging from the “will expectation” concept to the “should expectation” concept to the “ideal expectation” concept. The *will* expectation concept focuses on forecasting or predicting future performance and is refers to a customer’s beliefs of what will happen in the postpurchase period. The *should* expectation concept establishes a normative standard for performance and relates to what a customer believes would happen in the next service encounter. The *ideal* expectation concept is concerned with optimal performance and relates to what a customer wants in an ideal sense [19]. Nevertheless, under ideal expectation, it is theoretically unsound to assume that performance levels that exceed the ideal standard result in higher perceived quality than performance levels that are equal to the ideal standard. Furthermore, if expectation is interpreted to represent a feasible ideal, a positive monotonic linkage between the perception-expectation measure and perceived quality would not be expected when the attributes involved are finite ideal attributes [21]. Second, the concept of expectations ignores the possibility that consumers’ expectations change as a consequence of consumers’ experience and the impact of changes on subsequent cognitive processes [15]. Third, the concept of satisfaction construct is also ambiguous. Some authors view satisfaction as an attitude (eg, [22]), while others differentiate satisfaction from attitude (eg, [23]).

To tackle these limitations, we measured both preknowledge and postknowledge expectations in one model. While preacceptance expectation is based on secondhand experience (eg, others’ opinions or information disseminated through mass media), postacceptance expectation is formed by the customers’ firsthand experience and is more realistic [15]. In Bhattacherjee’s [15] study, this postacceptance expectation is represented as perceived usefulness. Perceived usefulness can be viewed as individual belief or sum of belief, in that perceived usefulness is a cognitive belief salient to IS use [15,24]. Perceived usefulness is the only belief that consistently influences user intention across temporal stages of information systems use; thus, it is an adequate expectation in the information systems usage context [15]. Perceived usefulness is an important variable affecting users’ postadoption decisions since, in this stage, users are likely to reevaluate their early acceptance decisions and make their decisions about continued usage [19].

## Methods

### Research Model

**Figure 1:**
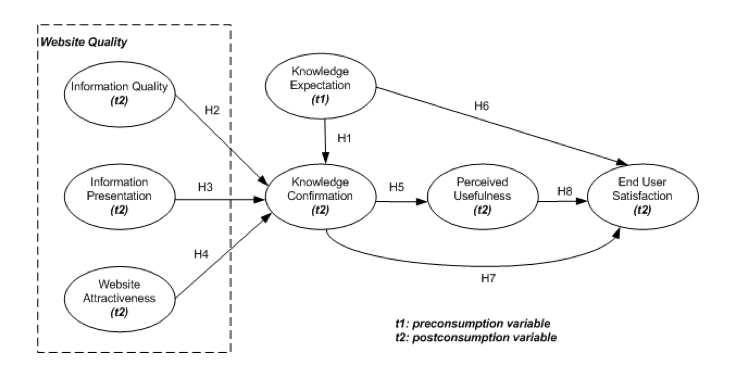
Research model.

The conceptual model that presents the hypothetical relationships is illustrated in Figure 1. This model shows how knowledge expectations and perceived website quality (ie, information quality, information presentation, and website attractiveness) can influence knowledge confirmation, which leads to perceived usefulness and end user satisfaction. Even though the original concept of expectation confirmation theory strongly suggests perceived performance as an antecedent of satisfaction, we are likely to use *website quality* rather than perceived performance as a predictor of knowledge confirmation. The website’s performance in delivering information can be dependent on the quality or nature of the information [25]. To this extent, expectation and confirmation measures focus on personal knowledge or skills, while website quality measures focus on the technical aspects of information. On the basis of ECT discussed previously, we define knowledge expectation as a preconsumption variable (labeled t1 in Figure 1), and the remaining variables as postconsumption variables (labeled t2 in Figure 1). The description of each construct is presented in in Table 1.

**Table 1:** Definitions of constructs

Construct	Definition
Knowledge expectation (Adapted from [14] )	Customers’ existing attitudes or beliefs regarding expected levels of knowledge they may gain by accessing the website
Knowledge confirmation (Adapted from [15])	A cognitive belief (the extent to which user’s knowledge expectation of information systems use is realized during actual use) derived from prior information systems use
Information quality [26]	Quality of the information system output
Information presentation [27,28]	The degree to which information presentation effectively facilitates interpretation and understanding
Website attractiveness [28,29]	Website’s graphic style, that is, the tangible aspect of the online environment that reflects the “look and feel” of the website
Perceived usefulness [24]	An individual’s salient belief that using the technology (website) will enhance his or her job performance
Consumer satisfaction [30]	The summary psychological state resulting when the emotion surrounding confirmed or disconfirmed expectations are coupled with the customer’s prior feelings about the consumption experience

### Hypothesis Development

With respect to expectations as comparative referents, it is argued that this expectation influences the confirmation paradigm [31]. In this study, we assumed knowledge expectation to be a user’s *will* expectation. Unlike prepurchase behavior conceptualized in marketing concepts, under eHealth services, users have developed positive *will* expectations prior to information access [32]. To support this hypothesis, we have adopted motivation theory (expectancy-value theory) [31] and cognitive consistency theory [33]. First, according to basic motivation theory, motivation may be rooted in the basic need to minimize physical pain and maximize pleasure, and expectancy-value theory suggests that individual expectancies for success and the value they place on achieving it are important determinants of their motivation to perform different achievement tasks [31]. In this case, their positive expectation influences achievement or performance behavior [31]. Second, from a cognitive consistency perspective, the desire of actors to maintain cognitive consistency should affect how they interpret any perceived failures in their counterpart’s performance [34]. According to Lord and Maher [33], because actors are more likely to maintain cognitive consistency, those who report high initial expectation will use this as a basis for interpreting the behavior of their counterpart, including the extent to which they determine whether their expectation is met. Similarly, Joyce and Piper [32] have shown that patient expectancy variables are strong predictors of therapy outcomes. Following this prior literature, we also hypothesized that higher knowledge expectation will lead to higher knowledge confirmation (that is, met expectations). Thus, hypothesis 1 is that knowledge expectation is positively associated with knowledge confirmation.

Perceived quality may represent perceived performance of a product or service [20]. Quality assessment relative to expectation represents the most pervasive perspective on quality [35]. From the point of view of expectation, quality is defined by conformance to customer expectation that may relate to excellence, value, and other attributes that are salient to consumers in shaping their perceptions of quality [35]. The perceived level of quality may either confirm or disconfirm preexpectation. Furthermore, satisfaction is positively affected by expectation and the perceived level of disconfirmation. To this extent, if disconfirmation is perceived to have occurred, then customer satisfaction increases or decreases from the initial expectation [15].

Chiu et al [16] also found that perceived quality is the main predictor of quality confirmation. In our study, we defined three aspects of a website that need to be considered to evaluate knowledge-intensive websites. These are: (1) information quality, (2) information presentation, and (3) website attractiveness. Rogers [36] described information as a difference in matter-energy that effects the uncertainty in situations where a choice exists among a set of alternatives, that is, as matter-energy, information can travel through many forms and channels. In this case, information on the quality of health care is crucial for patients to make informed decisions, and the availability of this information will further empower patients in their relationship with physicians [4]. Information itself contains both extrinsic and intrinsic value that may shape perceptions of quality in the context of use [35]. Following this definition, Nelson et al [35] categorized information quality into four core dimensions: accuracy, completeness, currency, and format. Accuracy refers to the degree to which information is correct, unambiguous, meaningful, believable, and consistent; completeness is the degree to which all possible states relevant to the user population are represented in the stored information; currency represents the degree to which information is up-to-date; and format refers to the degree to which information is presented in a manner that is understandable and interpretable to the user [35].

Bliermel and Hassanein [3] investigated customers’ use of the Internet to locate and evaluate health-related information for self-learning, and the result indicated that content quality and technical adequacy played significant roles. Gallant et al [10] investigated the desire content and functionality from the patient-consumer perspective on a hospital website and suggested that website attributes such as visual elements, well-organized personalized information, quality information and reputation, and user-centric design are the important factors to develop eHealth websites. In order to provide a positive user experience, usable technology and the presentation and design of information should be considered as critical factors of website design [10,37]. Jiang and Benbasat [37] examined the effects of various online product presentation formats on consumers’ product understanding by specifying two indicators of product understanding performance: consumers’ actual product knowledge and perceived website “diagnosticity.” The results of this study suggested that the lack of Internet interface to present detailed product information likely leads to customers being less knowledgeable and less informed in making their decision. Thus, we hypothesized the following: (1) hypothesis 2: information quality is positively associated with knowledge confirmation; (2) hypothesis 3: information presentation is positively associated with knowledge confirmation; and (3) hypothesis 3: website attractiveness is positively associated with knowledge confirmation.

Liao et al [19] argued that confirmation during actual use will affect postconsumption expectations such as perceived usefulness. By adopting the concept of cognitive dissonance theory, Bhattacherjee [15] pointed out that users may experience cognitive dissonance or psychological tension if their preacceptance usefulness perceptions are disconfirmed during actual use. Rational users may try to remedy this dissonance by distorting or modifying their usefulness perceptions in order to be more consistent with reality.  Thus, confirmation will tend to elevate users’ perceived usefulness, and disconfirmation will reduce such perception. Moreover, Jiang and Benbesat [37] posited that the actual knowledge gained by users will positively influence the perceived usefulness of the website. Thus, we hypothesized that knowledge confirmation is positively associated with perceived usefulness (hypothesis 5).

The direct relationship between expectation and customer satisfaction has been proposed in prior research (eg, [14]). According to Bhattacherjee [15], the direct relationship between expectation and satisfaction can be explained by adaptation level theory, which posits that human beings perceive stimuli relative to or as a deviation from an adapted level or baseline stimulus level, where this adapted level is determined by the nature of the stimulus, the psychological characteristics of the individual experiencing that stimulus, and the situational context. The higher the expectation is, the higher one’s satisfaction with the service or product, and, conversely, the lower the expectation, the lower one’s satisfaction. Thus, we hypothesized that knowledge expectation has a positive effect on end user satisfaction (hypothesis 6).

Confirmation is positively associated with satisfaction as it implies realization of the expected benefits of information systems use, while disconfirmation (to the extent where perceived performance lags expectation) indicates failure to achieve expectation [15]. Through content analysis, Lewis [38] suggested that the use of technology may improve patients’ knowledge, involve them in health care decisions, and in turn, lead to better health outcomes. She also posits that the key concern is how to understand the way patients process information and translate it into action. If we can evaluate the best way to deliver the message/information, we will better understand how to use technology to optimize its advantage as a health care learning resource. Major empirical findings also support a positive relationship between expectation and satisfaction (eg, [17,20,30]). Thus, we hypothesized that knowledge confirmation is positively associated with end user satisfaction (hypothesis 7).

Perceived usefulness is the main reason that people decide to use and accept new information systems [10]. Determining the elements of online health information retrieval experience and incorporating those elements in websites that are deemed to contain high quality information from a medical expert’s perspective may lead to customer satisfaction [3]. The relationship between perceived usefulness and consumer satisfaction has also been shown by previous studies (eg, [15]). Thus, we proposed that perceived usefulness has a positive effect on end user satisfaction (hypothesis 8).

### Measurement of Variables

As far as possible, items used to measure each construct were based on preexisting instruments, and some of these were modified specifically for this study. Information quality items were adopted from Wixom and Todd [39]. We modified items developed by Rai et al [27] and Zhang and von Dran [28] to measure information presentation, and the items for website attractiveness were adapted from Montoya-Weiss et al [29] and Zhang and von Dran [28]. Items for expectation were based on Khalifa and Liu [17]; however, in our study, users were asked to recall the time when they first accessed the website. Moreover, questions for confirmation and end user satisfaction were adopted from Bhattacherjee [15] and Oliver [14], while questions for perceived usefulness were modified from Davis [24] in that respondents were asked to evaluate four forms of information (e-learning, e-book, PowerPoint and multimedia, and testimonial/Q&A format). This research instrument (questionnaire) was checked by academic professors from the information systems department, and a pretest was conducted to ensure the item measures were well communicated and understood. The items used in this study are presented in Multimedia Appendix 1. 

### Sample and Research Procedure

Our research used the website satisfaction survey, conducted by the National Cancer Center in South Korea. The survey applied a national probability sampling methodology to assess Korean residents’ perceptions regarding cancer information and other issues delivered by National Cancer Center. The objective of this survey was to measure customer satisfaction and identify the effectiveness of media usage to distribute the cancer-related information. The questionnaire was administered online by posting the electronic form on the NCC (National Cancer Center) website. When users entered the website, the questionnaire was presented on a new browser window (pop-up window). Data were collected from September 18, 2009 through December 28, 2009. Cash rewards were provided for respondents. Upon the completion of this survey, 200 responses had been collected. In the present study, we excluded data from respondents with an elementary school education level as our *t* test suggested that there was a significantly different perception between this group and the other groups [40], resulting in a study sample of 198 usable responses.

Of the 198 respondents, 71.2% (141) were female. The majority of respondents (100) were from 20 to 29 years of age (50.5%), while 52 were from 30 to 39 years of age (26.3%). More than half (67.7% or 134) of respondents had a university degree. Among the 198 respondents, 47.5% (94) obtained information about cancer information from the Internet, 19.7% (39), from television, 8.6% (17), from family, 8.1% (16) from a medical center, and the remaining 16.2% (32) obtained information about cancer from friends, books, cancer clubs, newsletters, hospital instructions, and other resources. The percentages of respondents that heard about the NCC website by word of mouth and through Internet searches were 44.4% (88) and 40.9% (81) respectively, while others learned of the website from various other sources (eg, brochures, newsletters, advertisements, and recommendations). Furthermore, among the respondents, approximately 59.6% (118) were members of the general population, followed by 22.2% (44) who were family members or other relatives of patients, 14.1% (28) who were researchers/academics, and only 4.0% (8) who were patients. Lastly, we also asked the respondents to indicate how the information they obtained was used. More than 70% (73.2% or 145) of respondents used the information as resource or reference material, while 26.3% (52) and 17.2% (34) used it as self-learning and to educate cancer patients, respectively.

## Results

### Reliability and Validity

Prior to data analysis, the research instrument was assessed for its reliability as well as its construct validity. Construct validity assessment was performed through confirmatory factor analysis (CFA) using LISREL 8.7 (Scientific Software International, Inc, Lincolnwood, IL). Each scale item was modeled as a reflective indicator of its latent construct. The seven constructs were allowed to covary in the CFA model. First, we checked the scale validity by examining the goodness of fit of the overall CFA model using criteria suggested by Choudhury and Karahanna [41], where the ratio of chi-square to degrees of freedom should not exceed 5; normed fit index (NFI), comparative fit index (CFI), and goodness of fit index (GFI) should be greater than .90; adjusted goodness of fit index (AGFI) should exceed .80; and root mean square error of approximation (RMSEA) should not exceed .80. After exclusion of some invalid items (the third item of information presentation and the first item of knowledge expectation), all indices of goodness of fit (χ^2^/df = 1.51; RMSEA = .051; NFI = .91; NNFI = .96; CFI = .97; GFI = .90; AGFI = .83) suggested an adequate model fit of the empirical data. Furthermore, convergent validity was evaluated using three criteria suggested by Fornell and Larcker [42]: (1) all indicator factor loadings should be greater than .70, (2) composite reliabilities (CR) should be greater than .80, and (3) average variance extracted (AVE) should exceed .50. All factor loadings exceeded .70. Composite reliabilities ranged from .84 to .93, and AVE ranged from .63 to .86 (see Table 2). Therefore, all three conditions for convergent validity were met. Lastly, discriminant validity was assessed using criteria recommended by Fornell and Larcker [42], where the square root of AVE should be larger than the correlation scores among constructs. The result indicated that the condition for discriminant validity was also met (see Table 3).

**Table 2:** Confirmatory factor analysis results

Variable and Item		Item Number	Standardized Solution	Construct Reliability	Average Variance Extracted
**Information quality**				.92	.68
	The website provides accurate information.	IQ1	.84
	The website provides up-to-date information.	IQ2	.74
	The website provides relevant information.	IQ3	.82
	The website provides the content that supports the website's intended purpose.	IQ4	.86
	The website consists of appropriate level of information detail.	IQ5	.88
**Information presentation**				.87	.63
	The overview, table of contents, and/or summaries/headings are clearly organized.	IP1	.79
	The structure of information presentation is logical.	IP2	.84
	The information presented is understandable.	IP4	.77
	The amount of information presented was just right.	IP5	.78
**Website attractiveness**				.93	.69
	Overall, the website's color use is attractive.	WA1	.87
	This website has visually attractive screen layouts.	WA2	.87
	This website has an attractive screen background and pattern.	WA3	.85
	This website has eye-catching images or title on homepage.	WA4	.82
	The multimedia contents are attractive.	WA5	.80
	This website is fun to explore.	WA6	.74
**Knowledge expectation**				.84	.72
	Using this website will increase my knowledge level about cancer-related subjects.	KE2	.87
	Using this website will improve my skills through a learning process.	KE3	.83
**Knowledgeconfirmation**				.92	.86
	I have learned new knowledge by using this website (as I expected).	KC1	.93
	I have improved my skills by using this website (as I expected).	KC2	.92
**Perceived usefulness**				.90	.69
	Web tutorial/e-learning	PU1	.89
	Tutorial material in a printable PDF file/e-books	PU2	.85
	PowerPoint slide presentation	PU3	.79
	Testimonial and Q/A content	PU4	.79
**Customer satisfaction**				.92	.86
	Considering all things, I'm very satisfied with this website.	SF1	.92
	Overall, my interaction with this website is very satisfying.	SF2	.93

**Table 3:** Discriminant validity

Variable	IQ	IP	WA	KE	KC	PU	SF
Information quality (IQ)	.83						
Information presentation (IP)	.72	.80					
Website attractiveness (WA)	.51	.65	.83				
Knowledge expectation (KE)	.59	.54	.48	.85			
Knowledge confirmation (KC)	.70	.69	.61	.62	.92		
Perceived usefulness (PU)	.52	.59	.45	.51	.54	.83	
User satisfaction (SF)	.57	.65	.71	.54	.61	.56	.92

We tested the possibility of common method bias by adopting Harman method bias [43]. A single factor explained 40.3% of the variance, indicating no evidence of common method bias. To strengthen our conclusion, we also adopted a marker variable technique recommended by Lindell and Whitney [44]. First, we chose one unrelated criterion variable (user frequency) to serve as the method variance marker variable. Next, we measured the estimation value of the correlation between the method variable and the manifest variables (r_s_). Then, we calculated the partial correlation scores (*r* *yi-M*) using equation 1 (see Textbox 1). We measured the confidence interval using the test statistic (equation 2). Furthermore, using equation 3, we estimated the scores corrected for unreliability and common method variance (CMV) (*r’* *yi-m*).

**Marker Variable Technique**

Equation 1:
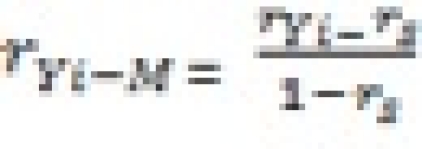

Equation 2:
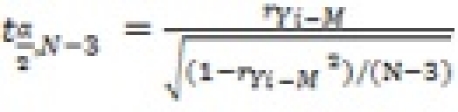

Equation 3:
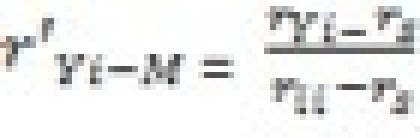

Where:

*r_yi-M_* is a partial correlation between variable X_i_ and Y, controlling for M (unmeasured relevant cause),

*r_yi_* is the correlation coefficient suspected of being contaminated by the common method variance (CMV),

*r_s_* is the correlation between the method variable and the manifest variable X_1_ multiplied by the correlation between the method variable and the manifest variable X_2_,

N is the number of samples,

*t* is the confidence interval,

*r’_yi-M_* is the disattenuated correlation between Y and M,

*r_Yi_* is an observed correlation coefficient, and

*r_ii_* is the reliability of X_i_.

Table 4 presents the correlation results of the measurement constructs. In the first model, we placed perceived usefulness as a dependent variable whereas information quality, information presentation, website attractiveness, knowledge expectation, and knowledge confirmation were predictors. In the second model, perceived usefulness was treated as a predictor of user satisfaction as presented in our original model. As can be seen in Table 4, all relevant predictors have statistically significant correlations with the criterion variable, whereas the theoretically irrelevant predictor has an insignificant correlation with the criterion variable. The correlations of the marker variable (MV) with other predictor variables are low, supporting the discriminant validity of the MV. All the correlation scores remain statistically significant even when CMV is controlled, revealing that these predictors account for theoretically meaningful amounts of the variance. Moreover, the application of equation 3 shows that the disattenuated partial correlations of all four variables with the criterion are slightly higher than the first-order partial correlations, indicating the unreliability always decreases the estimated impact of CMV [44].

**Table 4:** Hypothetical correlation among constructs (n = 198)

Variable	IQ	IP	WA	KE	KC	UF	PU	SF
IQ	.83							
IP	.72*	.80						
WA	.51*	.65*	.83					
KE	.59*	.54*	.48**	.85				
KC	.70*	.69*	.61*	.62*	.92			
UF	.19	.17	.08	.18	.21	1.00		
PU	.52*	.59*	.45**	.51*	.54*	.24	.83	
SF	.57*	.65*	.71*	.54*	.61*	.24	.56*	.92
*ryi-M (PU)*	.50*	.57*	.44**	.49**	.52*	.00		
*r’yi-M(PU)*	.54*	.66*	.47**	.59*	.56*	.00		
*ryi-M (SF)*	.55*	.64*	.70*	.52*	.59*	.00	.53*	
*r’yi-M(SF)*	.60*	.73*	.76*	.62*	.64*	.00	.60*	

*P < .05

**P < .01.

Therefore, we concluded that common method bias does not seem to be a serious problem in this study. Regarding multicollinearity, variance inflation factor (VIF) scores were measured for all constructs as in Gable et al [45]. The VIF scores ranged from 5.57 to 2.30, below the common VIF threshold of 10, indicating all items were subjected to further analysis [45]. Lastly, nonresponse bias was measured by verifying that the early and late respondents were not significantly different [46]. 

### Structural Model and Hypotheses Testing

**Figure 2:**
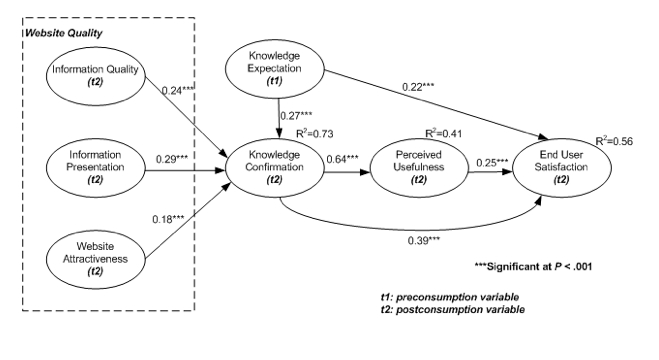
Hypotheses results.

The structural equation model was used to test the eight hypotheses proposed in this study (see Figure 2). All fit indices have suggested adequate model fit between the proposed model and the actual data (*X* *2* */df* = 1.72; RMSEA = .061; NFI = .90; NNFI = .94; CFI = .95; GFI = .90; AGFI =. 81). As we hypothesized, knowledge expectation exhibited a positive effect on knowledge confirmation (beta = .27, *P* *<* .001), accepting hypothesis 1. The paths from information quality, information presentation, and website attractiveness to knowledge confirmation were also positive and significant (beta = .24, *P* < .001; beta = .29, *P* < .001; beta = .18, *P* < .001 respectively). 

Thus, hypotheses 2, 3 and 4 were accepted. Moreover, the effect of knowledge confirmation on perceived usefulness was also positively significant (beta = .64, *P* < .001); thus, hypothesis 5 was also accepted. Knowledge expectation together with knowledge confirmation and perceived usefulness also significantly affected end user satisfaction (beta = .22, *P* < .001; beta = .39, *P* <.001; beta = .25, *P* <.001 respectively). Hence, hypotheses 6, 7, and 8 were accepted. The model explains 73% of the variance in knowledge confirmation, 41% of the variance in perceived usefulness, and 56% of variance in end user satisfaction.

## Discussion

### Principal Results and Limitations

Korea has one of the most advanced information technology and IT infrastructure in the world, supporting the diffusion of eHealth technology not only domestically, but also outside the country. Therefore, eHealth has become one of the most important elements for public health care, health informatics, and other related technologies in South Korea. As one of the initial public health care services in this country, the National Cancer Center, initiated by the Ministry of Health and Welfare, South Korea, also delivers its services through the Internet. One of the main functions of this website is providing cancer information in various forms, including electronic learning, e-books, multimedia presentations, and testimonials [47]. As cancer is a common cause of death and its rate is expected to increase in Korea [47], the effort to empirically study the method of delivering health information in Korea may contribute to both research and practice as we proposed in the previous section.

First, our empirical research showed that knowledge expectation was positively associated with actual knowledge confirmed by users after accessing the eHealth website (ie, met expectation). Unlike the traditional ECT in marketing research, our findings confirmed that higher preknowledge expectation may lead to higher postknowledge confirmation. We argued that users or patients’ expectations motivate them to access the website, with the assumption that they will gain more knowledge. This finding also supports Joyce and Piper’s [32] findings, indicating that initial expectation is a strong predictor of learning outcomes.

Second, this study also found that website quality (ie, information quality, information presentation, and website attractiveness) also influenced the actual knowledge confirmation. Additionally, from our survey, online searching for cancer information is the most popular choice for obtaining information compared with other conventional alternatives. Grounded on this finding, we argued that computer-based information has been an effective strategy for knowledge transfer in the health care context [38]. Moreover, it indicated that as potential patients, website users want to get functional, interactive, and efficient information, that is, knowledge-intensive websites might be the key to enhancing the likelihood of people using health care websites. The website attributes of information presentation and attractiveness are also needed to stimulate learning effectiveness, thus increasing the actual knowledge confirmation [37].

Third, the findings confirmed the positive relationship between knowledge confirmation and perceived usefulness (postexpectation variable) suggesting that users’ perceptions of the usefulness of information provided by an eHealth website may be influenced by their confirmation level. Considering the fact that this confirmation level was influenced by website quality, we argued that when the expectation and information quality attributes are both measured in the preconsumption stage, postexpectation is related to information quality [48]. Furthermore, we showed that the usefulness of a website is also be supported by a better design of the website to meet user needs [10].

Fourth, the effects of knowledge expectation, knowledge confirmation, and perceived usefulness on end user satisfaction were also statistically significant. Through these findings, we posit that user satisfaction is determined by expectation of the knowledge and confirmation of expectation following actual use represented by perceived usefulness [15]. Users form this expectation distribution based on their cumulative expectation through postconfirmation, influencing their further perception. To this extent, however, confirmation also showed a greater effect than both preexpectation and postexpectation constructs, confirming the findings of Bhattacherjee [15] and Oliver [49]. The results also suggested that confirmation may influence satisfaction directly or through the mediation of perceived usefulness, indicating that the relationship between confirmation and satisfaction levels can be modeled in two different ways: using both direct and mediation effects.

Beyond its contribution, this study also has limitations. First, we only investigated the predictor side of satisfaction. Further research is needed to study the outcome side of the satisfaction model (eg, the relationship between satisfaction and intention to use and the relationship between satisfaction and negative word of mouth). Second, even though a range of statistical methods has been used to ensure the validity and reliability of our data, further research is needed to measure the expectation and confirmation at adoption and postadoption to validate the results. Third, this study was based in Korea and used only one specific cancer website. Future research can explore the importance of information and knowledge for different respondents in different countries.

### Implications and Future Research

This study provides implications for both research and practice. Theoretical implications of this research are threefold: (1) identification of the attributes of knowledge-intensive websites; (2) enhancement of the theoretical foundation of eHealth from the information systems perspective by adopting ECT; and (3) examination of the importance of information and knowledge and explanation of their impact. First, the raising of concerns about the validity of information on the Internet has been a challenge for eHealth centers whose goal is to provide knowledgeable information presented in an appropriate format and posted on an interactive website. Our study also suggests that an intensive website should be able to influence the cognitive skills of users in learning and absorbing knowledge. Further research may address this initial finding to study how the website attributes presented in this study together with other attributes (eg, service quality) influence consumers’ attitudes in a different sense.

Second, this study has enhanced the concept of electronic health care from the information systems perspective by providing theoretical explanations through the adoption of ECT. By demonstrating that preknowledge expectations and perceived information performance influence actual knowledge acquisition, the results indicate that when patients and or users enter a health care website, they bring a certain level of expectation that by accessing and turning on the website, they may improve and gain some new information and knowledge, while explicitly, this process is also influenced by perceived performance. We also argued that during the consumption process, the user’s expectations might be adjusted by confirmation, resulting in greater or lower postexpectation beliefs (perceived usefulness). Thus, our study suggests the important linkage of these variables for eHealth satisfaction literature. We measured preexpectation with *will* expectation and the confirmation results showed the *met* expectation condition. However, by considering the ambiguity of the original expectation concept, future research should examine this theory in greater depth.

Third, this study examined the online information performance construct by studying its effects on influencing the knowledge cognition process. Recognizing that transfer of knowledge to patients or end users may help them to participate in the decision-making process toward their health condition, we suggest that further research is needed to examine the roles of other information media, such as mobile information services. Moreover, it is also a challenge for information systems researchers to become involved actively in this area, particularly to examine how to deliver health information in various electronic formats.

Practically, our empirical results indicate that information performance is a core requirement for knowledge building. Through this study, we argued that having accurate, high quality cancer or general health care information published on a reliable website can provide individuals with knowledge and help the consumers to obtain more useful materials. Furthermore, this research suggests that information on eHealth websites should be presented attractively. Online health care can also provide an opportunity for health care centers to learn how to provide online information innovatively to attract more patients or Internet users. The information presentation in various formats (eg, multimedia/power point and e-book) can utilize multiple sensory channels to convey information to users, which, in turn, builds respective mental representations in both verbal and nonverbal system [50]. Thus, eHealth providers should consider these attributes to build their knowledge-intensive websites. Another implication is related to consumer satisfaction. Our study pointed out that confirmation has a greater effect on satisfaction than other variables. Thus, it is not sufficient for a health care center to just improve its quality information attributes, it must also try to meet patients’ expectation, and in turn, increase their actual confirmation. 

## Multimedia Appendix 1

Measurement Items.

## Abbreviations

AGFI: adjusted goodness of fit index
AVE: average variance extracted
CFA: confirmatory factor analysis
CFI: comparative fit index
CMV: common method variance
CR: composite reliability
df: degrees of freedom
ECT: expectation confirmation theory
GFI: goodness of fit index
IP: information presentation
IQ: information quality
IS: information systems
KC: knowledge confirmation
KE: knowledge expectation
MV: marker variable
NCC: National Cancer Center
NFI: normed fit index
PU: perceived usefulness
RMSEA: root mean square error of approximation
SF: satisfaction
UF: user frequency
VIF: variance inflation factor

## References

[1] Bates BR, Romina S, Ahmed R, Hopson D (2006). The effect of source credibility on consumers' perceptions of the quality of health information on the Internet. Med Inform Internet Med.

[2] Sciamanna CN, Clark MA, Houston TK, Diaz JA (2002). Unmet needs of primary care patients in using the Internet for health-related activities. J Med Internet Res.

[3] Bliemel M, Hassanein K (2006). Consumer Satisfaction with Online Health Information Retrieval: A Model and Empirical Study. e-Service Journal.

[4] Altinkemer K, Prabuddha D, Zafer O (2006). Information system and health care XII: toward a customer-to-healthcare provider (C2H) electronic marketplace. Communications of the Association for Information Systems.

[5] Maheu MM, Whitten P, Allen A (2001). E-Health, Telehealth, and Telemedicine: A Guide to Start-up and Success.

[6] Eysenbach G (2001). What is e-health?. J Med Internet Res.

[7] Eysenbach G, Powell J, Kuss O, Sa ER (2002). Empirical studies assessing the quality of health information for consumers on the world wide web: a systematic review. JAMA.

[8] Jang Z, Chan J, Tan B, Chua W (2010). Effects of interactivity on website involvement and purchase intention. Journal of the association for information system.

[9] Hwang H-G, Hung M-C, Lin M-H, Yen DC, Chang I-C (2007). Factors affecting the adoption of electronic signature: Executives' perspective of hospital information department. Decision Support Systems.

[10] Gallant L, Cynthia I, Kreps G (2006). User-centric hospital websites: A case for trust and personalization. e-Service Journal.

[11] Ketchum AM (2005). Consumer health information Websites: a survey of design elements found in sites developed in academic environments. J Med Libr Assoc.

[12] Fitterer R, Rohner P, Mettler T, Winter R (2010). A taxonomy for multi-perspective ex ante evaluation of the value of complementary health information systems-applying the unified theory of acceptance and use of technology.

[13] Anderson EW (1994). Cross-category variation in customer service and retention. Marketing Letters.

[14] Oliver RL (1980). A cognitive model of the antecedents and consequences of satisfaction decisions. Journal of Marketing Research.

[15] Bhattacherjee A (2001). Understanding information systems continuance: an expectation-confirmation model. MIS Quarterly.

[16] Chiu C-M, Hsu M-H, Sun S-Y, Lin T-C, Sun P-C (2005). Usability, quality, value and e-learning continuance decisions. Computers & Education.

[17] Khalifa M, Vanissa L (2003). Determinants of satisfaction at different adoption stages of Internet-based services. Journal of the Association for Information Systems.

[18] Huang GJ, Penson DF (2008). Internet health resources and the cancer patient. Cancer Invest.

[19] Liao C, Chen J-L, Yen DC (2007). Theory of planning behavior (TPB) and customer satisfaction in the continued use of e-service: An integrated model. Computers in Human Behavior.

[20] Rust RT, Inman JJ, Jia J, Zahorik A (1999). What you don't know about customer-perceived quality: the role of customer expectation distributions. Marketing Science.

[21] Teas K (1993). Expectations, performance evaluation, and customers' perception of quality. The Journal of Marketing.

[22] LaTour S, Peat N (1979). Conceptual and methodological issues in consumer satisfaction research. Advances in Consumer Research.

[23] Hunt H, Hunt H (1977). CS/D-overview and future research directions. Conceptualization and Measurement of Consumer Satisfaction and Dissatisfaction.

[24] Davis F (1989). Perceived usefulness, perceived ease of use, and user acceptance of information technology. MIS Quarterly.

[25] McKinney V, Yoon K, Zahedi FM (2002). The measurement of Web-customer satisfaction: an expectation and disconfirmation approach. Information Systems Research.

[26] DeLone W, McLean E (2003). The DeLone and McLean model of information systems success: A ten-year update. Journal of Management Information Systems.

[27] Rai A, Lang SS, Welker RB (2002). Assessing the validity of IS success models: An empirical test and theoretical analysis. Information Systems Research.

[28] Zhang P, von Dran GM (2000). Satisfiers and dissatisfiers: A two-factor model for website design and evaluation. Journal of the American Society for Information Science.

[29] Montoya-Weiss MM, Voss GB, Grewal D (2003). Determinants of online channel use and overall satisfaction with a relational, multichannel service provider. Journal of the Academy of Marketing Science.

[30] Oliver RL, Linda G (1981). Effect of satisfaction and its antecedents on consumer preference and intention. Advances in Consumer Research.

[31] Wigfield A (1994). Expectancy-value theory of achievement motivation: A developmental perspective. Educational Psychology Review.

[32] Joyce AS, Piper WE (1998). Expectancy, the therapeutic alliance, and treatment outcome in short-term individual psychotherapy. J Psychother Pract Res.

[33] Lord R, Maher K (1993). Leadership and Information Processing: Linking Perceptions and Performance.

[34] Adobor H (2005). Trust as sensemaking: the microdynamics of trust in interfirm alliances. Journal of Business Research.

[35] Nelson R, Todd P, Wixom B (2005). Antecedents of information and system quality: an empirical examination within the context of data warehousing. Journal of Management Information System.

[36] Rogers E (1995). Diffusion of Innovations.

[37] Jiang Z, Benbasat I (2007). The effects of presentation formats and task complexity on online consumers' product understanding. MIS Quarterly.

[38] Lewis D (1999). Computer-based approaches to patient education: a review of the literature. J Am Med Inform Assoc.

[39] Wixom BH, Todd PA (2005). A theoretical integration of user satisfaction and technology acceptance. Information Systems Research.

[40] Wilson E, Balkan S, Lankton N (2010). Current trends in patients' adoption of advanced e-health services.

[41] Choudhury V, Karahanna E (2008). The relative advantage of electronic channel: A multidimensional view. MIS Quarterly.

[42] Fornell C, Larcker D (1981). Evaluating structural equations with unobservable variables and measurement error. Journal of Marketing Research.

[43] Harman H (1976). Modern Factor Analysis.

[44] Lindell MK, Whitney DJ (2001). Accounting for common method variance in cross-sectional research designs. J Appl Psychol.

[45] Gable G, Sedera D, Chan T (2003). Enterprise systems success: a measurement model. Proceedings of the International Conference on Information Systems.

[46] Armstrong J, Overton T (1977). Estimating nonresponse bias in mail surveys. Journal of Marketing Research.

[47] National Cancer Control Institutue, National Cancer Center (2009). Cancer Facts and Figures 2009 in the Republic of Korea.

[48] Oliver RL, Burke RR (1999). Expectation processes in satisfaction formation. Journal of Service Research.

[49] Oliver R (1993). Cognitive, affective, and attribute bases of the satisfaction response. Journal of Customer Research.

[50] Jiang Z, Benbasat I (2007). The effects of presentation formats and task complexity on online consumers' product understanding. MIS Quarterly.

