# Supplementary material for: Website Quality, Expectation, Confirmation, and End User Satisfaction: The Knowledge-Intensive Website of the Korean National Cancer Information Center
Source: J Med Internet Res. 2011 Nov 2;13(4):e81. doi: 10.2196/jmir.1574 (PMC3222204; doi:10.2196/jmir.1574)
Supplement: Supplementary file 1 [file jmir_v13i4e81_app1.pdf]

Multimedia Appendix 1. Measurement Items

Variable	Items
**Information Quality**	IQ1. The website provides accurate information
	IQ2. The website provides up-to-date information
	IQ3. The website provides relevant information
	IQ4. The website provides the content that supports website's intended purpose
	IQ5. The website consists of appropriate detail level of information
**Information Presentation**	IP1. The overview, table of contents, and/or summaries/headings are clearly organized
	IP2. The structure of information presentation is logical
	IP3. The objectives of the module are clearly presented (dropped)
	IP4. The information presented is understandable
	IP5. The amount of information presented was just right
**Website Attractiveness**	WA1. Overall, the website's color use is attractive
	WA2. This website has visually attractive screen layouts
	WA3. This website has attractive screen background and pattern
	WA4. This website has eye-catching images or title on homepage
	WA5. The multimedia contents are attractive
	WA6. This website is fun to explore
**Knowledge Expectation**	Back to your first visiting to this website, please indicate your Cognitive/Knowledge expectation:
	KE1. I would find this website useful in my purpose(s) (dropped)
	KE2. Using this website will increase my knowledge level about cancer related subject
	KE3. Using this website will improve my skills through learning process
**Knowledge Confirmation (met expectation)**	After using this website, please indicate your actual Knowledge confirmation:
	KC1. I have learned a new knowledge by using this website (as I expected)
	KC2. I have improved my skills by using this website (as I expected)
**Perceived Usefulness**	In order to achieve your purpose(s), how useful do you think each of the following material presented on the website?
	PU1. Web tutorial/e-learning
	PU2. Tutorial Material in a printable PDF file/e-books
	PU3. PowerPoint slide presentation
	PU4. Testimonial and Q/A content

**End-user Satisfaction**	SF1. By considering all things, I'm very satisfied with this website
	SF2. Overall, my interaction with this website is very satisfying
